# The Shape of Medical Devices Regulation in the United Kingdom? Brexit and Beyond

**DOI:** 10.5204/lthj.3102

**Published:** 2023-11-21

**Authors:** Muireann Quigley, Laura Downey, Zaina Mahmoud, Jean McHale

**Affiliations:** University of Birmingham, United Kingdom; University of Liverpool, United Kingdom; University of Birmingham, United Kingdom

**Keywords:** Medical devices, regulation, patient safety, innovation, framework regulation

## Abstract

The United Kingdom’s *Medicines and Medical Devices Act* (MMD Act) 2021 received royal assent on 11 February 2021. In its passage through parliament, as well as in the accompanying Explanatory Notes, the Act was framed by the government as a necessary post-Brexit bill. Yet prior to this, it was widely presumed that existing statutory instruments, enacted in 2019 to address medical devices regulation in anticipation of a ‘No Deal Brexit’, would provide the United Kingdom (UK) with the necessary legal framework through the transition period and beyond. The European Union (EU) exit legislation included provisions aimed at aligning domestic law with the new EU Medical Devices Regulation (EU MDR) and In Vitro Devices Regulation (EU IVDR) (which were initially due to be implemented during the EU exit transition period). However, just over a year later, at the end of 2020, while the 2021 Act was in the final stages of its parliamentary journey, legislation was introduced that reversed the provisions of the domestic medical device regulations concerning alignment for Great Britain (GB: England, Wales, and Scotland). The result is that the UK now has a dual system of regulation, with Northern Ireland (NI) being governed by the new EU MDR and EU IVDR and GB by older pre-Brexit (yet still EU-derived) law.

This article is an attempt to analyse the complex situation now in existence regarding the regulation of medical devices in the UK. To this end, we focus on three main issues. First, we examine the difficulties and challenges presented by the dual system of regulation between NI and GB, highlighting the potentially far-reaching consequences of regulatory divergence between different UK jurisdictions. Second, we ask whether, in the rush to new legislation in 2021, opportunities to properly reform the approach to medical devices were missed. Finally, we look to the future, focusing on the recent Medicine and Healthcare products Regulatory Agency Consultation on the future of medical devices regulation in the UK and the challenges and opportunities that remain.

## 1 Introduction

The United Kingdom’s (UK) *Medicines and Medical Devices Act* (MMD Act) was passed in early 2021, gaining royal assent nearly a year after it was somewhat suddenly introduced to parliament in February 2020. We say ‘somewhat suddenly’ because, although a Medicines and Medical Devices Bill (MMD Bill) was mentioned in the Queen’s Speeches at the opening of parliament in 2019 (which happened twice in quick succession that year),^1^ its appearance so early in the agenda was surprising given that legislation amending the UK’s regulations relating to both medicines and medical devices had only recently been passed in April 2019.^2^ These 2019 Regulations dealt with the UK’s (at the time) pending exit from the European Union (EU). In the specific case of medical devices, the purpose of these EU Exit Regulations was both to ensure that the UK’s Medical Devices Regulations 2002 were ‘fit for purpose in a no deal EU Exit scenario’ and to ‘mirror (insofar as that is possible) all the key elements’ of forthcoming (at the time) EU regulations relating to medical devices.^3^ Crucially, they ensured that the 2002 Regulations continued to have a legal basis post-Brexit, as they implemented primary EU law and were originally created using powers conferred by section 2(2) of the *European Communities Act 1972*, which would no longer apply after exit day. The 2019 Regulations also created limited powers to amend the Regulations where necessary to mitigate the effects of Brexit. With the immediate legal problems created by Brexit for the medical device regulatory regime solved by the 2019 Regulations, the urgency for further reform or amendment seemed reduced. Yet, in early 2020, the new Bill was introduced in the House of Commons. By the end of 2020 new EU Exit Regulations were passed amending aspects of the 2019 Regulations.

According to the Queen’s Speech of December 2019, the purpose of the MMD Bill was to ‘ensure that our NHS and patients [could] have faster access to innovative medicines, while supporting the growth of our domestic sector’.^4^ The concern, as articulated in the Explanatory Notes accompanying the Bill in February 2020, was to ensure that the UK continued to have a primary legislative instrument through which to update its regulatory frameworks pertaining to medicines and medical devices at the end of the Brexit transition period. Just shy of a year later, the MMD Act became the first piece of primary legislation on medical devices in the UK post-Brexit (indeed, ever). Moreover, it was being touted (by the government) as an opportunity to future-proof the regulatory regime and to clarify and improve the existing framework. In this article, we provide the first indepth critical analysis of the new Act, its place in the broader medical devices regulatory landscape in the UK, and the potential changes to this landscape that future regulations, consequent on the Act, might bring.

To these ends, we start in section 2 by explaining the development of the existing legal regulation of medical devices in the context of its recent reforms. We will argue that medical devices regulation has become overly complex and burdensome, not least because, as we are about to see, the UK now operates a dual system of regulation between Northern Ireland (NI) and Great Britain (GB: England, Wales, and Scotland). Following this, in section 3, we scrutinise this new system, arguing that the regulatory divergence between the different UK jurisdictions is problematic, with the potential to become even more so over time. In section 4, we interrogate the MMD Act itself, asking what opportunities it presents, or indeed misses, for real and substantive reform. Finally, in section 5, we look towards the UK’s possible regulatory future. Here, we examine the recent Consultation on the future of medical devices regulation by the UK’s Medicines and Health products Regulatory Agency (MHRA) and the government response to this. We will see that many of the proposed changes look very similar to those contained in the 2019 EU Exit Regulations, mirroring, as they did, the relevant changes in EU legislation. Given this, we query what could be considered an imprudent use of time and public resources to return us to something akin to the 2019 GB status quo. However, we also argue that there are some welcome changes in the pipeline, although only time will reveal the ultimate success of these as the UK forges a new path outside its closest major trading block.

## 2 Whither Medical Devices Regulation in the United Kingdom?

As we noted in the introduction, the MMD Act is the first piece of *primary* legislation to be enacted in the UK pertaining to medical devices. Until 2021, medical devices in the UK were governed solely by secondary legislation—the Medical Devices Regulations 2002—that implemented three EU directives: Medical Device Directive 93/42/EEC (MDD), Active Implantable Medical Devices Directive 90/385/EEC (AIMDD), and In Vitro Diagnostic Medical Device Directive 98/79/EC (IVDD). However, although it is a landmark piece of legislation—in the sense of being the first piece of domestic primary legislation in this area—we will see that the 2021 Act is a framework Act. The substance of medical devices regulation remains, for now at least, located in the 2002 Regulations. Given this, let us begin with an overview of the 2002 Regulations and the impact of Brexit on them before moving on to examine the 2021 legislation. We explore the changes that have occurred and their timeline. These changes speak to the complexity of the current medical devices law wrought by the proliferation of legislation. This is relevant to understanding the potential shape of future legal regulation, which we discuss in section 5 of this article.

### 2.1 The Medical Devices Regulations 2002: A Tale of Two Regulatory Regimes

The 2002 Regulations, as they existed prior to Brexit, were voluminous and had, over the years, been subject to a number of technical and other amendments, and their complexity has only been increased by the Brexit-related amendments. As already noted, they originally implemented the three EU Medical Devices Directives (MDD, AIMDD, and IVDD), setting out the relevant definitions (such as ‘medical device’, ‘manufacturer’, ‘place on the market’, and so on), alongside the requirements for devices falling under their ambit. The Regulations follow a risk classification system, with devices being covered by one of four risk classes: Classes I, IIa, IIb, and III. They also set out the essential requirements that devices must meet, for instance, those relating to the safety and performance of devices and the need for clinical evidence.

The EU Directives that underpinned the UK Regulations had long been acknowledged to be out of date, struggling, for instance, to accommodate technological advances such as developments in software engineering and the use of standalone software applications as medical devices.^5^ Additionally, there had been a number of scandals relating to medical devices within the EU, most notably a series of failures relating to the Poly Implant Prothèse (PIP) breast implants (which were more prone to rupture than other implants and contained unapproved silicone gel) and metal-on-metal hip replacements (which had a much higher revision rate than other types of hip replacements).^6^ Consequently, around the time Brexit was taking place, two new EU Regulations—which tightened up multiple aspects of the regulatory framework—were due to replace the older Directives: the EU Medical Devices Regulation 2017/865 (EU MDR) and the In Vitro Diagnostic Regulation 2017/866 (EU IVDR). Initially, these two EU Regulations would have been fully implemented in May 2020 and May 2021, respectively. However, the COVID-19 pandemic delayed their implementation by a year, meaning that the EU MDR came into force in 2021 and the EU IVDR in 2022.

The *European Union Withdrawal Act 2018* made provision for any EU law that was operative prior to 29 March 2019 to be retained in the UK as domestic law. However, while negotiations had been ongoing between the UK and the EU in 2019, there was some concern that the UK could leave the EU without a legal agreement. Consequently, provisions were put in place to address this eventuality. To this end, in relation to medical devices, the Medical Devices (Amendment etc.) (EU Exit) Regulations 2019 were passed.^7^ These Regulations put in place provisions to address issues that would have arisen if no agreement was reached between the EU and the UK, but where the UK was dependent on EU agencies and databases. Intriguingly, they also made provision for the implementation of the new EU MDR and EU IVDR despite the fact that such alignment was not legally required. The EU MDR and EU IVDR would not have been in effect before this date and, therefore, would not automatically have been retained. The 2019 Regulations, which had been subject to consultation on the MHRA website, thus continued regulatory alignment with the approach that was taken in the EU.^8^

Ultimately, there was not a ‘no deal’ Brexit, but equally, neither the EU MDR nor the EU IVDR was fully in force across the EU at the time of the UK’s eventual exit in December 2020. During the extended implementation period granted by the *European Union (Withdrawal Agreement) Act 2020*, the 2019 Regulations were also amended by the Medical Devices (Amendment etc.) (EU Exit) Regulations 2020.^9^ These Regulations continued the approach of the previous Regulations, providing for continuity in the case of retained EU law. However, in contrast to the 2019 approach, these later Regulations removed the provisions enabling the implementation of the EU MDR and EU IVDR for GB, with the EU Regulations only applying in NI. This latter aspect was required due to the Northern Ireland Protocol (as part of the Withdrawal Agreement), which came into effect on 1 January 2020.^10^ The Protocol set out the arrangements agreed upon between the UK Government and the EU on the position of NI post-Brexit, including the requirement that EU legislation relating to medical devices continue to apply in NI. Yet, by choosing to rescind the 2019 Regulations and not mirror the EU changes in GB, the UK Government created a dual system of regulation between NI and GB. Further, in June 2021, the Medical Devices (Northern Ireland Protocol) Regulations 2021 were laid before parliament with the aim of implementing the EU MDR in NI, something that could not be achieved through direct applicability of the EU MDR alone.^11^

These legislative developments need to be seen alongside the government’s stated ambition for the UK to become a destination for innovation across the medical technology (MedTech) sector.^12^ This is illustrated, for example, by a new Innovative Devices Access Pathway, the pilot of which has now launched with applications going live on 25 September 2023.^13^ This is a joint initiative between the MHRA and a number of partner organisations, including the National Institute for Health and Care Excellence, Health Technology Wales, and the Scottish Health Technology Group to create a new regulatory pathway ‘to bring innovative technologies and solutions to the forefront of the National Health Service (NHS), through a new, integrated support service for developers’.^14^ The initial information available about the pathway provides that the ‘MHRA can now look beyond the confines of the Medical Devices Directive to consider new regulatory opportunities’.^15^ We will return to discuss this new initiative below when examining the future shape of UK medical devices regulation. For now, we simply note that it is clear that there is aspiration at the state level to (re)frame post-Brexit UK as an innovation hub. This desire is explicitly set out elsewhere; for example, the webpage of the Department for Business and Trade states that ‘[t]he UK is a prime location to identify medical technology innovation and to research, develop and evaluate products and services in the world-renowned NHS’.^16^ It even permeates the wording of the new MMD Act, as we discuss below.

### 2.2 The Medicines and Medical Devices Act: An Act of Little Substance?

At the same time that the 2020 Regulations were passed, rescinding the earlier changes mirroring the EU MDR and EU IVDR (December 2020), the MMD Bill was still being debated and was about to enter the final stages of its passage through parliament. Significantly, an examination of Hansard from the time reveals that the updated Regulations passed without a single comment on the implications of this policy reversal.^17^ Indeed, it appears to be the case from some comments in the House of Lords, in particular, that members believed that 2019 Regulations aligning the UK with the EU would still be operational.^18^ Although there are aspects of the new Act to be welcomed, as we demonstrate in this section, overall, it potentially opens the door for the ever-increasing complexity and proliferation of regulations in this area, as well as compounding the problem of inadequate parliamentary scrutiny of derivative secondary legislation.

First, we should note that the 2021 Act deals with three distinct areas: medicines, veterinary medicines, and medical devices. While there are some parallels between the more general provisions in each of these areas, our focus is on these as they apply to medical devices. In this respect, as we indicated earlier, the MMD Act can be seen as a framework Act. Its primary purpose is to confer regulation-making powers upon the Secretary of State, enabling them to amend or supplement the 2002 Medical Devices Regulations.^19^ It also sets out some general principles that must guide these powers. Hence, section 15 states that:

(2) In making regulations under subsection (1), the Secretary of State’s overarching objective must be safeguarding public health.
(3) In considering whether regulations under subsection (1) would contribute to this objective, the Secretary of State must have regard to—
  (a) the safety of medical devices;
  (b) the availability of medical devices;
  (c) the likelihood of the United Kingdom being seen as a favourable place in which to—
    (i) carry out research relating to medical devices,
    (ii) develop medical devices, or
    (iii) manufacture or supply medical devices.^20^

Within this, we can see the clear attempt to position the UK as a ‘favourable place’ in which to develop medical technology post-Brexit. This wording was amended from the Bill as originally introduced, which instead referenced ‘the attractiveness of the UK as a place in which to develop or supply medical devices’.^21^ Additionally, the Bill as introduced did not reference the safeguarding of public health, although the government had passed an amendment by the end of the Lords Committee stage to require that in making regulations, the Secretary of State be ‘satisfied that they would promote the health and safety of the public’.^22^ However, in doing so, they would still have regard for the safety of devices, their availability, and the attractiveness (as it was then written) of the UK, as highlighted above.^23^ These aspects were rightly criticised in debate. It was noted in the House of Lords debates that the concept of attractiveness is ‘vague and open to misunderstanding’.^24^ There was a concern that without an explicit requirement to take the health and safety of the public into account, the attractiveness requirement might undermine these aspects.^25^ Following pressure from the Lords, additions regarding health and safety were made, and attractiveness was replaced by a favourability requirement. The former are certainly positive additions, but it is questionable whether ‘favourable’ is any different from, let alone better than, ‘attractive’ in the context of the Act.^26^ Either way, the desire to portray the UK as a MedTech- and innovation-friendly destination comes through strongly.

The regulation-making powers under section 15 of the 2021 Act are wide-ranging. They include making provisions for regulations to be made regarding the requirements for devices to be marketed; the ‘design, manufacture, composition or other characteristics of the devices’; assessments relating to medical devices (including evidence requirements); ‘the packaging of medical devices, and information, labelling or instructions’; establishing medical device registers, evaluations of safety and performance; and market surveillance.^27^ However, these provisions are merely enabling. They empower the Secretary of State to make new regulations rather than introducing substantive provisions in and of themselves. We say ‘merely’, but they are far from trivial since the Act enables large and widespread changes to this key policy area to be made via secondary legislation at some later date. What this might consist of will be examined later in this article. Whatever the exact shape of these might be, arguably, the use of delegated powers in this manner is inadvisable and will likely lead to inadequate scrutiny of future provisions.

There is no doubt that the introduction of secondary legislation is a significantly speedier process than the passing of primary legislation, and this can be of benefit when dealing with fast-paced policy areas such as MedTech. Nevertheless, there are serious concerns about delegating a major area of public policy in such a manner. This was put succinctly by Alex Norris MP during debate in the Commons, who said:

The proposed arrangements allow the Secretary of State and his successors to make hundreds or more individual decisions to change our current regulatory regime into a markedly different one, one statutory instrument at a time, which I do not think is desirable.^28^

It is for these reasons, among others, that the House of Lords Delegated Powers and Regulatory Reform Committee stated that ‘skeleton legislation should only be used in the most exceptional circumstances’.^29^ Further, the House of Lords Select Committee on the Constitution was highly critical of the MMD Bill, stating:

This is a skeleton bill containing extensive delegated powers, covering a range of significant policy matters, with few constraints on the extent of the regulatory changes that could be made using the powers. The Government has not provided the exceptional justification required for this skeleton approach^30^.

In light of these concerns, members in both the Commons and Lords tabled a sunset clause amendment.^31^ However, concerns were expressed that a sunset clause would establish a worrying precedent that could extend to other legislation.^32^ Ultimately, the amendment was rejected. For us, this is regrettable. Such a clause would have enabled the government to continue to make changes and be responsive in the short-term, post-Brexit period. It would also have given them time to draft more substantive (and adequately scrutinised) primary legislation relating to each of the three important policy areas in the MMD Act (medicines, veterinary medicines, and medical devices). We set out some of the missed opportunities in not doing this in the next section. However, despite our criticisms of these matters, there are a number of provisions in the legislation that are welcome, although it remains to be seen how they will work out in practice. The first is that the Act makes provision for a Commissioner for Patient Safety (more generally referred to as a Patient Safety Commissioner (PSC)). As we will see below, this was a late addition to the Bill. Baroness Cumberlege tabled an amendment during the Lords debates on the Bill to require the government to create this role.^33^ This came soon after the publication of the Report of the Independent Medicines and Medical Devices Review, which Baroness Cumberlege chaired. This report detailed a catalogue of institutional and systems failures concerning sodium valproate, hormone pregnancy tests, and pelvic mesh, raising questions about how patients can be kept safe and how harm caused by such failures ought to be dealt with.^34^ The Commissioner’s core duties are to promote patient safety and ‘the importance of the views of patients and other members of the public in relation to safety’.^35^ We discuss this in more depth in section 4.

In addition to the creation of the role of PSC, the Act provisions include the establishment of information systems relating to the safety and performance of medical devices, the safety of individuals with a medical device, and the improvement of safety and performance.^36^ These provisions are to be welcomed in principle. As the Cumberlege Review highlighted, there is a pressing need for systems and processes that can track data relating to medical devices to better identify when things go wrong and to address—and ideally prevent—any harm. However, as elsewhere in the legislation, these are enabling provisions, and inevitably, the structure and success of any such systems will depend on their final form and implementation. Significantly, Part 3 of the Act also introduces important amendments regarding enforcement provisions, including criminal and civil provisions for the breach of obligations relating to medical devices. The existing Regulations are notable for their absence of such provisions and arguably, therefore, for lacking effective enforcement.

Having set out in broad terms the current regulatory landscape across the UK, in the next sections, we look at two related issues. First, in section 3, we examine more closely the dual system of regulation that now exists between GB and NI and the potential implications for the future of medical devices regulation in the UK. Second, against this backdrop, in section 4, we then consider some of the missed opportunities that, we argue, the MMD Act represents.

## 3 Medical Devices and Regulatory Divergence within the United Kingdom

As we have already seen, as the result of multiple EU Exit Regulations amending the 2002 Medical Devices Regulations, the UK now has a dual system of regulation for medical devices between NI and GB. The political backstory of how we have ended up in this situation has its roots in the Good Friday Agreement (part of the NI peace process),^37^ which required that there be no hard border with the Republic of Ireland. However, since the Republic of Ireland is part of the EU, this posed a dilemma for the UK in relation to Brexit. The Northern Ireland Protocol and subsequent Windsor Framework (both of which are largely aimed at ensuring smooth trade within the UK) attempt to address this while also honouring the terms of the Good Friday Agreement.^38^ The full details of these Brexit agreements are complex, not to mention (politically) controversial, and there is no space to set them out here. The main point, for our purposes, is to note that the Protocol (updated by the more recent Framework) sets out the EU legislation that would continue to apply in NI, including the legislation relating to medical devices.^39^.

In many ways, the systems between NI (and the EU) and GB remain similar. This is because the general principles of the EU Directives (which still basically underpin the GB system) and EU MDR and EU IVDR are broadly similar. Each sets out the essential requirements that devices in particular risk classifications must meet to be placed on the market. These include requirements pertaining to pre- and post-market monitoring and evidence of safety. To ensure that manufacturers comply with these requirements in the EU and the UK, medical devices are subject to conformity assessments. These assessments are carried out by private third-party bodies known as Notified Bodies in the EU and (now) Approved Bodies in the UK. The essential requirements and conformity assessment route that devices must meet are determined by their classification. The older MDD and AIMDD, as well as the EU MDR, follow a cascading rules system. Thus, as we saw earlier, medical devices are classified as Class I, IIa, Iib, and III. There are, however, some notable differences between the two systems.

The EU MDR and EU IVDR introduced more stringent essential requirements, changed the classification of certain devices, and established the Unique Device Identification (UDI) System. The new EU Regulations have also tightened the provisions on equivalence, which allow the existence of devices already on the market to be used as evidence of a new device’s safety where they are deemed to be ‘equivalent’.^40^ This rule, along with the lack of traceability of medical devices, had been severely criticised following the metal-on-metal and PIP scandals.^41^ Tightening the equivalence provision and developing the UDI system were part of efforts to address this criticism and increase safety. Additionally, under the IVDD, and thus, in GB, there are no classifications per se, but there are categories for devices that indicate the specific assessment route to be followed.^42^ Meanwhile, in the EU, the IVDR has established a new classification system for in vitro diagnostic devices.

A significant point of divergence between NI and GB is that there are now different routes to, and conformity marks for, devices being placed on the market depending on where goods originate and where they are destined for. Within the EU and, before Brexit, across the UK, all medical devices placed on the market were required to be affixed with a Conformité Européenne (CE) mark. The purpose of this is to indicate that the goods in question (medical devices in this case) have met the requisite health and safety standards. Manufacturers are responsible for ensuring that devices comply with the Regulations to gain a CE mark, which is obtained from the Notified Body that conformity-assesses the device. There are different Notified Bodies located all over Europe, and successfully going through the conformity process with one of these is enough to gain access to the entire EU market. As part of the Brexit process, the UK Government has introduced the UK Conformity Assessed (UKCA) mark, and they intend this to be required for all devices placed on the GB market. This process, however, has been far from smooth, with the rules and timelines on this changing even at the time of writing.^43^

It is the UK Government’s intention that, eventually, medical devices to be placed on the GB market will require a UKCA mark and will need to apply to a UK Approved Body to carry out a conformity assessment of their devices.^44^ Devices intended for the NI market will require either a CE mark^45^ by pursuing a conformity assessment with an EU Notified Body^46^ or a UKNI mark with a conformity assessment carried out by a UK-based conformity assessment body.^47^ The UKNI mark is always to appear with a CE mark and is thus sometimes referred to as a CE UKNI mark. However, unlike devices awarded CE marks from EU Notified Bodies, devices bearing the UKNI/CE UKNI mark will not be able to be placed on the EU market.^48^ However, to gain such a mark, the device will need to be conformity assessed by a designated body (to be called a ‘UK Notified Body’). To date, however, no conformity assessment bodies have received the requisite designation.^49^ In the meantime, Regulations introduced in 2023 have extended the transitional periods so that CE-marked devices compliant with either the medical device Directives or the EU MDR/IVDR can be placed on both the GB and NI markets.^50^ This is to ease the significant post-Brexit regulatory administrative burdens for manufacturers and other economic actors.

One consequence of this is that NI may come to occupy somewhat of a privileged position in terms of the development and manufacture of medical devices.^51^ This is because the structure of the dual system of regulation means, in essence, that manufacturers who assemble and process their devices in NI or whose devices are in ‘free circulation’ in NI can access both the NI and GB markets. Consequently, devices placed on the NI market that have either a CE mark or a UKNI mark may gain entry to the GB market if they are a ‘qualifying Northern Ireland good’.^52^ Moreover, manufacturers based in NI whose devices qualify and who pursue conformity assessment with an EU Notified Body and receive a CE mark can access not only the whole of the UK market but also the EU market. Whilst it is not yet clear what arrangements will be in place once the transitional period for CE-marked devices ends, the government has committed to ensuring ‘unfettered access’ to the GB market for businesses and manufacturers established in NI.^53^ In theory, this could be a boon to medical device manufacturers based in NI compared to those in GB.

Having said this, the situation is a bit more complex from the perspective of the NHS and patients in NI and GB. As Hervey and colleagues point out, NI receives most of its medical devices from the EU.^54^ Since NI is, in effect, part of the EU for the purposes of trade relating to medical devices, this means that the supply of those devices to the NHS and, thus, patients in NI is reasonably straightforward. The complexities and complications with the implementation of the EU MDR and EU IVDR, which all Member States are contending with, notwithstanding, NI looks like it will continue to get its medical devices from the same place—the EU—as it always has done. This also means that once CE marks are no longer recognised in GB, where new or innovative devices are authorised in the EU, NI could gain access to such devices before the rest of the UK. In principle, the NHS in NI could also have access to devices from the mainland. However, the vagaries of the UKNI marking system set out above mean that this may not happen in practice. While goods for the GB market will eventually need a UKCA mark, this cannot be used for goods destined for NI. These, as we noted, will need either a CE mark from an EU Notified Body or a UKNI/CE UKNI mark from a UK Notified Body (where they are not destined for the EU market). If this remains the case, then after the transitional arrangement regarding CE marking expire, medical devices from GB will de facto not be able to be placed on the NI market unless they get conformity assessed by an EU Notified Body.

## 4 The Medicines and Medical Devices Act 2021: Missed Opportunities for Reform

In section 2, we saw that, as a piece of framework legislation, the MMD Act is light on substantive provisions. These are due to be incorporated in future statutory instruments and/or regulatory guidance. We argued that this is problematic since it may lead to inadequate scrutiny of what could, in essence, be substantive changes to major policy areas (medicines, veterinary medicines, and medical devices).^55^ We will return in section 5 to discuss what these potential future regulations and policy changes might comprise. However, before we do this, we examine how, in developing the 2021 Act, there were a series of missed opportunities for law reform regarding medical devices. Specifically, there is (in our view at least) a pressing need to consolidate legislation regarding medical devices, and the MMD Act was an occasion to address this. We also see that while the Act does introduce some much-needed provisions for safeguarding the health of the public and patient safety, arguably, it does not go far enough.

### 4.1 Medical Devices Legislation: Fragmented and Unwieldy

As discussed in section 2, by the time the MMD Bill was laid before Parliament in early 2020, the medical devices regulatory regime had already become voluminous and complex. The 2002 Regulations implemented three different EU Directives and had been amended by several technical and other regulations over time, even before the passing of the 2019 EU Exit Regulations. The 2019 Regulations introduced a myriad of changes spanning well over 100 pages. There is a reasonable argument to be made that regarding medical devices before Brexit, the complexity of the legislation was not a cause for great concern. A look at the—albeit long—list of amending regulations shows that most were mainly technical—concerning, for example, changes to fees for medical devices registration and so on.^56^ Nevertheless, these changes had proliferated over time. For this reason, during the passage of the Bill, amendments were tabled in both the Commons and the Lords that would have required the government to bring forth consolidating legislation within a few years of the MMD Act’s passing.^57^ The Lords, in particular, pushed the government on this issue, with several Lords supporting such an amendment.^58^ There was support in principle from the government side for consolidation ‘as an opportunity to simplify and clarify’, although there was no agreement that an obligation to do this should be included in this particular Bill.^59^ Eventually, the amendment was withdrawn in favour of a government-tabled compromise provision requiring a report on the function of the Act five years after royal assent. On this, section 48 explicitly states that:

(2) The report must, in particular, include an assessment of whether—
  (a) some or all medicines and medical devices legislation should be consolidated or otherwise restructured,
  (b) provisions of medicines and medical devices legislation should be included in regulations or Acts of Parliament, and
  (c) powers to make regulations should be modified or repealed^60^.

Both Houses of Parliament accepted this position and, thus, withdrew the amendment containing the more concrete obligation to bring forth consolidating legislation.

The need for more streamlined legislation for medical devices has been made clear on a number of occasions since the Bill was originally introduced.^61^ Indeed, the need for this was something that a number of members of the government seemed to agree upon during debate on the Bill. However, there was some disagreement at the time about the best way to achieve this. One suggestion with some merit was that rather than an obligation to consolidate being built into the eventual Act, the Law Commission could be tasked with reviewing existing legislation and making recommendations.^62^ Whilst agreeing that the Law Commission would be best placed to take this on, the Government Minister in the Lords, Lord Bethell, said that he was not in a position to give any assurances that they would.^63^ Arguably, the need for consolidation and streamlining has become even more apparent in the short time since the Bill was debated and the Act passed.^64^ During the passage of the Act and subsequently, the regulatory landscape for medical devices has become even more fragmented and complex, with no fewer than seven statutory instruments relating to medical devices coming into force since 2020 (including the EU Exit Regulations passed during the latter stages of the MMD Bill’s journey).

The 2020 EU Exit Regulations rescinding the earlier 2019 changes to incorporate the EU MDR and EU IVDR framework in GB and creating the dual system of regulation in the UK, are, as we have already argued, problematic. More than this, however, they serve as a singular illustration of the dangers of both an overly complex regulatory landscape and an over-reliance on the use of delegated legislation. For us, it is concerning that these Regulations seemingly passed without much debate or extensive consideration. They were discussed at a formal meeting of the Delegated Powers Committee in November 2020. The meeting lasted 22 minutes, and only one opposition MP spoke on the matter.^65^ Introducing the 2020 Regulations, the Minister for Health stated that ‘[m]ost of the changes they make are technical in nature’.^66^ Yet a cursory look at the Regulations reveals that they go far beyond mere technical amendments. This represented a move away from the previously signalled policy of almost total alignment with the EU MDR and EU IVDR and did so with extremely limited Parliamentary scrutiny.

Additionally, the 2020 Regulations, plus other regulations since passed, add yet more layers of complexity to the existing regulatory regime. To fully understand the medical devices regime, the Regulations must be read together. Not only does this represent a barrier to understanding the legislation by those most affected by it—including device manufacturers, others with legal obligations under the Regulations, patients and end users—it also means that scrutiny of any changes to the regime can be challenging. This task has been improved somewhat by the long overdue publishing of an up-to-date version of the 2002 Regulations—with all amendments incorporated—on the official website (something that was not available when the MMD Act was proceeding through parliament).^67^ Nevertheless, with future widescale changes set to continue to be made by regulation using the powers of the 2021 Act, the already convoluted and complex array of amendments and cross-references will become increasingly hard to follow. In introducing primary legislation in the form of the MMD Act, there was an opportunity to deal with the existing regulatory complexity across three major policy areas. Even if an effective consolidation exercise would not have been possible in the timeframe in which the government were operating at the time, the provisions in the Act could have more concretely enabled such an exercise in the short to medium term following the passing of the Act. In terms of the obligations under section 48 of the Act, since a report on the operation of the Act is only due before the end of five years, we are not far enough into the legislative cycle to see how they will play out. Conceivably, the tenor of the report will depend on the political will of the incumbents at the time the report is due (before February 2026).

Concerns over the appropriateness of the delegated powers and (in)adequate scrutiny of any resulting legislation formed the golden thread across multiple objections to the MMD Bill as it progressed through Parliament. But, of equal import throughout proceedings, and inextricably linked to this central theme, was the (lack of) prominence of patient safety within it.

### 4.2 Medical Devices and Patient Safety

Medical devices regulatory discourse is frequently characterised by twin objectives, which at times sit in tension with each other: (1) patient safety and (2) considerations of innovation and access to markets. Arguably, the balance between these too often falls in favour of industry, something that has demonstrably failed patients. This can be seen with large medical device scandals, such as the PIP breast implant and metal-on-metal hip scandals referred to earlier, as well as the fact that recalls and warnings relating to medical devices have risen dramatically.^68^ In the UK, for example, there were 62,000 adverse incident reports linked to medical devices between 2015 and 2018.^69^ It is not surprising, therefore, that the Regulatory Horizons Council Report on Medical Devices in 2021 recommended that ‘[t]he regulation of medical devices should be centred on the needs of patients, informed by patients, record outcomes that matter to patients, and provide evaluations that are understandable to patients’.^70^ In making this recommendation, the Council highlighted themes found in the Cumberlege Review report, which had been published just a year before.^71^

As outlined in section 2, the Review was published in between the Commons and Lords debates on the MMD Bill and, as such, affected the shape of the subsequent Act. It brought with it significant political pressure for the government to respond to its recommendations, and the MMD Bill was seen as an opportunity to try to implement them. In particular, the recommendation to create a new PSC role as a way to improve patient safety was pursued vigorously during debate on the Bill, including by Baroness Cumberlege herself. Consequently, provisions relating to this not only made it into the subsequent Act^72^ but also became the headline provisions constituting Part 1 of the Act in its entirety. In so doing, the Act was subtly—or perhaps not so subtly—reframed from essential post-Brexit legislation to one intimately concerned with patient safety. Whether this reformulation of the purposes of the Act will be borne out, as some of us have previously argued, very much depends on the implementation of the provisions in practice.^73^ In July 2022, Dr Henrietta Hughes was appointed as the first PSC.^74^ While this is clearly a welcome development, we have some concerns regarding the scope of the role and the PSC’s (lack of strong) investigatory powers mandated in the MMD Act.

Despite the PSC headlining the 2021 Act (see the preamble of the Act on this), Part 1 is exceedingly brief, leaving most of the detail (such as it is) to Schedule 1. Part 1 is concerned solely with ‘establishment and core duties’ and, on this, section 2 states that:

(2) The Commissioner’s core duties are to—
  (a) promote the safety of patients with regard to the use of medicines and medical devices, and
  (b) promote the importance of the views of patients and other members of the public in relation to the safety of medicines and medical devices.

However, as Moore has argued, there are two main problems with these provisions. The first is that this employs an unduly narrow conception of patient safety, which ‘extends far beyond ensuring the safety of medicines and medical devices’.^75^ In support of this, she cites research that shows that diagnostic errors account for the majority of avoidable harm in primary care in England.^76^ She also cites reports on failures within the healthcare system that demonstrate that we ought not to conceive of patient safety in narrow terms. These are the reports detailing ‘conditions of appalling care’ at Mid Staffordshire NHS Foundation Trust^77^ and one on avoidable baby deaths at Shrewsbury and Telford Hospital NHS Trust.^78^ It is clear from these reports that patient safety often has nothing to do with device and medicine safety and everything to do with failures of the wider healthcare system, as well as of leadership and governance. Given this, Moore rightly questions the way the core duties of the PSC are set out in the MMD Act.

This brings us to Moore’s second main criticism, which is that the narrow focus of the role on medicines and medical devices means that many may be left without any means of making their voices heard. She says, ‘[b]y way of example, this means that a patient harmed because of a poor hospital discharge experience would not be able to raise this with the PSC’.^79^ A related issue is that provisions in Schedule 1 of the Act actually bar the PSC from investigating individual cases, although they may take them into account in the context of considering some general issue or other.^80^ This is concerning, given how previous scandals have demonstrated that a lack of early action is a key factor leading to avoidable harm in the NHS. A properly functioning PSC could help facilitate the early identification of such harm and act as a ‘figure able to guide … and assist actors within the regulatory space in order to improve the system overall’.^81^ The emphasis here is, of course, on ‘properly functioning’. There is a significant concern that the PSC role mandated in the MMD Act has no real teeth. Schedule 1 of the Act, which contains the majority of the details of this new role, contains neither provisions regarding the scope of the investigatory powers of the PSC (apart from barring the investigation of individual cases) nor any with respect to enforcement or other powers to effect change. In this regard, the Act was a missed opportunity to put real statutory bite behind the PSC’s mandate.

Finally, it should be noted that the changes in relation to establishing the role of a PSC need to be read in conjunction with some other amendments that have a bearing on the issue more generally. The MMD Bill, as originally introduced, required that the Secretary of State have regard to three specific issues when making new regulations: the impact on patient safety, any impact on the availability and supply of medical devices (and medicines and veterinary medicines), and the likelihood of the UK being seen as an ‘attractive’ place to conduct clinical trials (in the case of medicines) and develop and market medical devices. Indeed, the attractiveness and availability clause were framed as the ‘twin pillars of the Government’s strategy on medicines and medical devices and the key themes of [the] Bill’.^82^ Leaving the EU, regardless of any eventual deal, was always going to have a negative impact on health care, generally speaking. This includes access to medical products.^83^ In this respect, while data on the percentage of medical devices supplied to the NHS from different jurisdictions is difficult to come by, we know that in 2021, imports of medical devices from the EU decreased to their lowest levels in years, though they recovered somewhat in the following months.^84^ The Regulatory Horizons Council Report on Medical Devices highlighted the need to increase the capacity of UK-based Approved Bodies to deal with the increased regulatory burden created by the introduction of the UKCA mark and to avoid the bottlenecks in the market that would otherwise occur.^85^ Hence, increased trade friction and costs created by Brexit will most likely have an impact on the costs of medical devices available on the NHS and, thus, on supply. Therefore, ensuring the availability of medical devices post-Brexit is critically important.

For us, at the time the Act was being debated, there were (and still are) unanswered questions about *how* the UK was to be made attractive post-Brexit outside the larger EU market, whether industry interests would be prioritised over the voices of patients and, fundamentally, whether safety standards would be traded off in a bid to make the UK market competitive.^86^ This was something that also concerned Parliament. Ultimately, amendments were passed that addressed the issue of safety by introducing a requirement that ‘the Secretary of State’s overarching objective’, when making regulations pursuant to the Act’s powers, ‘must be safeguarding public health’.^87^ Additionally, as outlined in section 2, the attractiveness clause was replaced by a requirement that the UK be seen as a ‘favourable place’ in which to develop, manufacture or supply medical devices.^88^ While there are open questions about how the three main considerations under this overarching objective—safety, availability, and favourability—are to be balanced and implemented in practice, it is encouraging that the eventual Act does include a so-called ‘lock on patient safety’.^89^ Specifically, it requires that where regulations may affect patient safety, the appropriate authority can only make them if it ‘considers that the benefits of doing so outweigh the risk’.^90^ In spite of this, although the addition of the overarching objective of safeguarding the health of the public may be an improvement, the Act arguably bestows upon the government an overly broad range of discretionary powers. Any new regulations introduced, as argued in section 3, could dramatically alter the landscape with respect to medical devices. This is concerning given that, among other things, this could have an outsized impact on patient safety and will largely be exercised under conditions of inadequate scrutiny. With this in mind, in the final substantive section of this article, we consider the potential shape of future medical devices regulation in the UK.

## 5 The Future of Medical Devices Regulation?

As we have observed throughout this article, medical devices regulation across the UK has been in a period of flux, something that is set to continue for some time into the future. From September to November 2021, the MHRA undertook a Consultation on the future of medical devices regulation. The government’s response was published in 2022.^91^ Together, the Consultation and the government response indicate the direction of travel for the UK. The proposals made cover a range of much-needed changes, many of which will contribute to the objectives set out in the MMD Act of safeguarding the health of the public, ensuring and improving patient safety, and enabling innovation. As a preliminary point, we note that the proposals are intended to cover the UK as a whole, including NI. The UK-wide rules are planned to ‘run in parallel’ with EU rules in NI, and goods (including medical devices) conforming to these rules can circulate in GB and NI.^92^ For clarity, however, in what follows, when we talk of future changes to the Regulations, we mean changes in comparison to the law as it currently applies in GB. In this respect, the MHRA proposals, largely accepted by the government, include extending and altering the scope of GB’s medical devices regulations, tightening up the devices classification system, making changes to the requirements for clinical investigation and performance studies, introducing measures to improve vigilance and (post-)market surveillance, and introducing new regulatory pathways and alternative routes to market. The Consultation was extensive, so here we focus on certain key issues. As we shall see, several of the proposed changes look strangely familiar.

Future regulations will extend the scope of existing regulations to include products without an explicit intended medical purpose if they have a similar risk profile to medical devices.^93^ Specific products listed include contact lenses, dermal fillers, devices involving brain stimulation (e.g., transcranial direct current stimulation), and diagnostic tests for health and wellbeing (e.g., genomic testing).^94^ This is a positive step, but this is not the first time we have seen this proposal. The specific products to be included within the scope of the new regulations are, in essence, products found in Annex XVI of the EU MDR, which deals with devices without an intended medical purpose.^95^ Similarly, other proposed changes seem to be a simulacrum of the EU rules. For example, substantial proposals to amend rules relating to performance evaluations and clinical studies, including more detailed requirements relating to informed consent and the circumstances in which pregnant women and minors can be included,^96^ are similar to those found in the EU MDR.^97^.

Also, closely following changes made in the EU MDR, there is a clear commitment to tightening up the devices classification system, with the rules regarding a number of matters, such as conformity assessment and the requirements for clinical evidence, set to change. Under current operations, the classification rules are different depending on device class; that is, whether devices are low-risk (Class I) or high-risk (Class III). Therefore, while a clinical evaluation (distinct from a clinical investigation or trial) must be completed for all devices to assess whether they conform to the requirements of the 2002 Regulations, manufacturers can self-certify the lowest-risk devices for the purposes of conformity marking. This means that while they must complete a clinical evaluation for low-risk devices, they do not need to submit this information for independent verification. Conversely, for the higher-risk devices, a conformity assessment by an Approved Body is required, which entails the submission of clinical evidence as to the safety and efficacy of their product. This evidence might be a clinical investigation of the device itself or evidence that a device is ‘equivalent’ to one already on the market.

The MHRA’s proposals would lead to stricter rules regarding the different risk classes. These changes would, in effect, lead to an up-classification of certain devices; for example, surgical mesh and closed loop systems (e.g., automated insulin delivery pumps) would become Class III devices rather than Class IIb. As with the EU MDR, this would bring with it more stringent requirements in terms of what is required for the clinical evaluation of devices. This would mean that, when these changes come into effect, devices that previously would have benefited from a lesser regulatory burden will now be subject to greater scrutiny, making the conformity assessment process more labour-intensive. This may be welcome in light of the significant harm that some devices, such as surgical mesh, have caused in recent years. In these instances, requiring greater evidence of safety for these devices by up-classifying them seems like an appropriate response to the harms caused. However, it is worth noting that while implementing more stringent regulatory standards can reduce or prevent some harms, there may also be unintended consequences. For instance, Melvin and colleagues have recently argued that a constellation of factors relating to the requirements of the EU MDR has decreased the economic viability of bringing to market or continuing to market devices used for certain rare conditions.^98^ While this issue is on the radar of various stakeholders within the EU, including the European Commission,^99^ it was not mentioned in the MHRA Consultation. However, it seems to us that this is an important issue to consider in relation to any future changes in the UK.

In any case, many of the UK’s proposed changes are, as just indicated, in line with the changes to classification brought about by the EU MDR. While this may seem to indicate future regulatory alignment between GB and the EU,^100^ any such alignment is destined to be incomplete. The indications are that we will have some interesting divergences from the EU approach, with the government keen to emulate positive practices from other jurisdictions and strive for global harmonisation on a number of aspects relating to medical devices. A notable and explicit divergence from the EU’s approach can be seen in the proposals relating to the use of ‘device equivalence’ as a route to market authorisation. Whereas the EU and the United States (US) both have equivalency routes, these only require that a device be similar to one with existing market approval, meaning in practice that equivalence is claimed where only a part of a device is similar to another.^101^ As we noted in section 3, the EU MDR has tightened the equivalence provisions (in comparison to the earlier MDD), setting out that ‘technical, biological and clinical characteristics shall be taken into consideration for the demonstration of equivalence’.^102^ This provides greater detail and a more holistic approach to determining the level of equivalence needed to rely upon a predicate device (the device to which equivalence is claimed). In essence, this has raised the bar for proving equivalence and reduced the range in variation of a device that can qualify for equivalency. Nevertheless, it leaves open the potential that devices need not be identical to rely on the new provision. Thus, the current rules still mean that there can be differences between the device for which authorisation is sought and the device for which equivalence is being claimed.^103^ The UK Government, however, plans that device manufacturers will need to demonstrate ‘entire equivalence’, meaning that similarity to only part of a device would not qualify.^104^ The government response states that ‘[t]his approach would take us beyond the equivalence requirements in the EU MDR’.^105^ While this sounds intriguing, at the time of writing, ‘entire equivalence’ has not been fully defined, and further information is not available as to what it might require of manufacturers.

Another departure can be seen in the move towards a lifecycle approach to the regulation of medical devices. This is closer to the ‘total product life cycle’ (TPLC) approach taken in other jurisdictions, most notably by the US Food and Drug Administration (FDA). Such an approach attempts to take an integrated view across the entire lifecycle of a device, including by using pre- and post-market (surveillance) data.^106^ This is most evident in the MHRA’s plans for software and artificial intelligence.^107^ Regarding this, the regulator emphasises the importance of reforms across the device lifecycle^108^ and proposes, for example, adopting predetermined change control plans (PCCPs) for software as a medical device Since software can—indeed, often must—change to implement improvements or updates, it is necessary to make provisions within the regulatory system to accommodate this. PCCPs would enable developers and manufacturers to set out anticipated changes and procedures relating to these, thus ensuring that they do not need to go through a new approvals process each time this happens.

A further notable divergence can be illustrated with the example of Unique Device Identification–Device Identifiers (UDI–DIs).^109^ Several jurisdictions require the use of these, including both the EU and the FDA, and the International Medical Device Regulators Forum (IMDRF) has published UDI guidance.^110^ A UDI is a distinct numeric or alphanumeric code used to identify a medical device. Assigning a UDI to a device has many benefits, including aiding traceability by tracking devices through supply chains, identifying counterfeit devices, and making the recall of defective devices easier.^111^ Nevertheless, as noted by Nichols, while ‘global industries are complicated, and as UDI began to spread across the globe, it also created a new and unique issue of broadly similar, yet slightly differing, UDI requirements’.^112^ The UK has opted to use the Global Medical Devices Nomenclature (GMDN) system,^113^ as it ‘is the most widely used nomenclature system worldwide’.^114^ While this system is also used in the US by the FDA, for example, it is different to that used by the EU, which uses the European Medical Device Nomenclature (EMDN). The EMDN system is mandated by the EU MDR^115^ and IVDR^116^ and is required for devices registered on the EUDAMED database (the EU’s—still in progress—database of medical devices).^117^ The European Commission has stated that ‘[t]o the extent possible, [it] will map the EMDN to the … GMDN’.^118^ Nevertheless, there are differences between the systems. So, although the UK Government has committed to working towards ‘the introduction of a globally harmonised device identification and coding system’,^119^ it is not a fait accompli. Moreover, opting for the GMDN system when the UK’s nearest trading partner uses a different one raises questions about the regulatory (administrative) burdens for GB manufacturers. There are also as yet unanswered questions about how NI fits into this. NI manufacturers with products bound for the EU will need to use the EMDN system, but what about devices for the NI and/or GB markets? Will UDI–DIs from both the GMDN and EMDN be needed?^120^ This is perhaps some of what is in the process of being worked out by the regulator and other stakeholders.

Further significant changes pending to GB’s regulatory system are the plans for alternative routes to market. The main proposals in this regard are threefold. The first of these would see the UK become a full partner in the Medical Device Single Audit Program (MDSAP). This is an initiative spearheaded by the IMDRF, which involves multiple jurisdictions, including the EU, the US, Canada, and Australia. The purpose of the MDSAP is to permit an ‘Auditing Organization to conduct a single regulatory audit of a medical device manufacturer that satisfies the relevant requirements of the regulatory authorities participating in the program’.^121^ Under the MDSAP, an Auditing Organisation can conduct a single audit relating to a medical device manufacturer’s quality management system to assess compliance with the regulatory standards of the program’s participating organisations. The exact role that MDSAP audit reports would have within the new GB system is not yet clear. The government response merely states that ‘[t]he MHRA will require UK Approved Bodies to consider MDSAP assessments; however, adoption will be optional for manufacturers’.^122^ What we can say is that, as with the situation with the requirements of the EU MDR and EU IVDR,^123^ MDSAP assessments would not replace the conformity assessment process itself. This is because a manufacturer’s quality management system for devices is only part of what is assessed when granting market authorisations. However, the use of MDSAP may help speed up the approvals process and make things more efficient for manufacturers seeking approvals in multiple jurisdictions that are all participants in the MDSAP.

In a second, related move, market authorisations could be expedited even more under a proposed Domestic Assurance route. This would allow for a shortened approvals process for devices that have already received approvals elsewhere. One concern with this is that it could lead to inadequate scrutiny, whereby manufacturers seek approval from the regulatory authorities with the least stringent requirements and use this to gain access to the GB market. Again, it is difficult to say how this would work in practice until more information becomes available. However, there is some reassurance to be gained from the fact that the government response explicitly states that Approved Bodies will have the power to reject applications under any such route.^124^ In addition, it states that:

The changes the MHRA will be taking forward will also ensure the UK aligns with international best practice where those standards are superior to current standards, and they will introduce greater transparency of regulatory decision-making through updating the requirements that apply to Approved Bodies and increasing the consistency of conformity assessments, for example.^125^

Again, although this is positive, much remains to be seen on the exact details of the proposals.

The third proposed alternative route to market is a new regulatory pathway for innovative MedTech. Plans for an Innovative Devices Access Pathway (IDAP) are, as we noted in section 2, already advancing in the form of a joint initiative of multiple UK agencies launched at the end of September 2023. Under this scheme, according to the government, ‘a key feature is that the MHRA would hold additional powers to grant initial market approval’.^126^ As envisaged in the Consultation document, this would see the MHRA grant market authorisation for a device before the manufacturer has obtained a conformity mark, although manufacturers would be required to switch back to the Approved Body route to obtain a UKCA mark. The idea is that the new pathway ‘creates a regulatory “sandbox” for innovators’, which will, in turn, facilitate the (quicker) introduction of innovative medical devices into the NHS.^127^ In granting the MHRA new powers to provide initial market approvals for innovative devices (as indeed they will also have for medicines in a parallel scheme: the Innovative Licensing and Access Pathway^128^), there are again questions that need to be asked about adequate scrutiny. Arguably, this is giving an already under-resourced regulator even more work to do, and if this is the case, then they may find themselves even more squeezed in terms of staff, time, and funding. The last UK budget did bring with it an announcement that the MHRA would receive an extra £10 m ‘to help bring innovative new medicines and medical technologies to UK patients more quickly’.^129^ Sometimes, however, budgetary promises do not work out, and this one was given against a backdrop of cuts at the MHRA in recent years. There has also been a significant increase in workload at the MHRA, consequent on the churn of regulatory changes with respect to both medicines and medical devices. Given this, even with such a large cash injection, it remains to be seen if it will be enough given the scale of the task ahead.

## 6 Conclusion

Medical devices regulation in the UK has been in a constant state of change for a number of years, even prior to the UK’s exit from the EU. Stakeholders across Europe, including regulators, Notified Bodies, and the MedTech sector as a whole, had been grappling with pending changes to the system. They had already spent considerable time and other resources preparing for the implementation of the EU MDR and EU IVDR. Adding to this, Brexit, the MMD Act, the global pandemic, and the delayed EU MDR implementation date came together to create a perfect regulatory storm. The result has been the layering of regulation upon regulation, leaving us with a fragmented, complex, and unwieldy corpus of law relating to medical devices. With even more new regulations and imminent policy changes planned, this is only set to get worse. In this article, we attempted to untangle and analyse some of the complexities and difficulties engendered by all of this. To this end, we examined the challenges presented by the dual system of regulation between NI and GB, the opportunities (missed and otherwise) presented by the MMD Act, and the shape that some of the future regulations might take given the recent MHRA Consultation and government response.

From our analysis, we can see that the (very particular) confluence of events that has led us to the current state of medical devices law and regulation will have far-reaching consequences. This is the case whether it is to be found in the regulatory divergence within the UK between NI and GB, the raft of secondary legislation that we now have (with more to come), or the many changes coming down the line that may see GB become a sort of a regulatory halfway house between the EU and other international approaches. However, whether or not the proposed changes will succeed in realising the government’s ambition either for the UK to become an innovation destination for medical devices or for improved patient and device-user safety remains to be seen. Much of the detail of the planned changes is not yet available. Nonetheless, whatever these turn out to be, there are a number of areas that will likely prove challenging for law- and policymakers as they try to navigate and implement the UK’s post-Brexit regulatory future.

A principal difficulty is the issue of regulatory divergence. In this article, we presented two contrasting sets of concerns in this respect. The first relates to the ongoing problem of how to avert worsening regulatory divergence between NI and GB. The government’s stated approach is to have a set of UK-wide rules, which are planned to ‘run in parallel’ with EU rules in NI.^130^ The appeal of this notwithstanding, we noted that—for the time being, at least—such a system seemingly privileges NI in terms of access for medical device manufacturers based there to both the GB and EU markets. This is in tension with the government’s drive to make the (whole of the) UK into an innovation hub. Beyond this, there are practical implications for patients and the NHS. It is possible that there may be situations where devices have received market authorisation in the EU but have not been approved in GB. The inverse may also be true. In the future, there may be devices that, depending on how the new market authorisation processes and access to market pathways pan out (e.g., the Domestic Assurance and IDAP routes), could receive market authorisation in GB but not in the EU. The UK Government’s ‘unfettered access’ provisions are, in effect, a one-way street. They are mainly driven by concerns about NI-based businesses accessing the GB market rather than by considerations of the supply of goods going into NI. They guarantee access to the GB market for devices bearing CE and CE UKNI marks, but not vice versa. The result of all this is that we could end up in a situation where medical devices can be placed on the market in one part of the UK but not another, potentially exacerbating issues of unequal access to (new) medical technologies across the UK.^131^

This leads us to the second set of concerns, which is that despite the general rhetoric regarding divergence from the EU and greater international alignment post-Brexit, the reality of the proposed changes indicates an uneasy mixture of both EU and international elements. As we saw, some of the proposed changes—such as extending the scope of the existing regulations to devices without an explicit intended medical purpose and tightening up the risk classification system—are not so new. They essentially mirror provisions in the EU MDR and EU IVDR and, as such, mirror changes originally written into UK law by the 2019 EU Exit Regulations before they were rescinded for GB by the 2020 Regulations. Sometimes, as the saying goes, the more things change, the more things stay the same.^132^ Where proposals do diverge from the EU—for instance, with the adoption of the GMDN system of device identification, a move towards a TPLC approach, the introduction of ‘entire equivalence’, and new pathways to market—they may not be entirely unproblematic. These measures, without more, do not guarantee the access to (and safety of) devices or the facilitation of innovation that the government is aiming for.

While the UK was part of the EU, it benefited from product harmonisation across and market access to *all* Member States. As Hervey and colleagues note:

Overall, the effects of EU law are to establish a relatively high regulatory barrier at the outside border of the EU, which must be met if products are to be sold in the EU. At the same time, EU law removes many non-tariff barriers to trade in medicines, medical devices and equipment *within* the EU, creating a single large market for which global providers of medicines, medical devices and equipment need to meet—and evidence—just one set of regulatory requirements in order to access the whole market.^133^

Being part of the EU meant not only that the UK could export products manufactured to other Member States in a relatively frictionless manner but also that the UK could import products from other Member States without the need to navigate the external trade and regulatory barriers that non-EU countries come up against. However, life outside the EU now means that GB has lost insider access to its largest trading partner. The consequence of this is an increase in the regulatory burden associated with accessing that market.^134^ The practical implications of this are that unless mutual recognition between the UK and other jurisdictions can be achieved (be they the EU, the US, or other international markets), or unless the UK offers special incentives to manufacturers, the gravitational draw of the larger EU and US markets will mean that the UK continues to be a less ‘favourable’ place in which to develop and supply medical devices.^135^ However, even if the UK does offer something like special incentives, there are important questions that will need to be asked about the consequences of doing so. For instance, if the planned international alignment fails and the new pathways to market are not successful enough to plug the gap left by the loss of (easy) access to the EU market, what options are left? Hervey and colleagues suggest that, after these, the UK may have to deregulate and lower standards in a bid to attract investment.^136^ We hope that it does not come to that. Such a move would not only run counter to the principled commitment to patient safety made in the MMD Act but also runs the risk of future medical devices scandals by disregarding past lessons, such as those from the PIP breast implant, metal-on-metal hip, and pelvic mesh scandals.

Whatever the eventual shape of medical devices regulation in the UK, what is certain is that we have not yet seen the end of this period of continuous regulatory change. We argued that future regulations could usher in radical policy changes that may not be subject to adequate scrutiny. The delegated powers contained in the MMD Act—and the process of regulation via secondary legislation—may have the benefit of allowing quicker and more responsive regulation of advancing technologies. However, this may come at the expense of more rigorous parliamentary oversight. Unlike primary legislation, secondary legislation does not allow for debate and scrutiny of individual provisions. Given this, and as we illustrated with the example of the 2020 EU Exit Regulations, there are serious concerns about delegating an area of public policy as important as medical devices to secondary legislation. This is especially so given that the repercussions of any policy change in this area could have an outsized impact on the health of, and potential harm to, the public. If the UK is to be successful as it pushes forward on its ‘new’ path outside its closest major trading block,^137^ then law- and policymakers will need to meet the challenges outlined in this article head-on.

## Footnotes

1 Queen Elizabeth II, “Queen’s Speech” October 14, 2019; Queen Elizabeth II, “Queen’s Speech December 19, 2019.”

2 Medical Devices (Amendment etc.) (EU Exit) Regulations 2019, SI 2019/791; Human Medicines (Amendment etc.) (EU Exit) Regulations 2019, SI 2019/775.

3 Explanatory Notes to the Medical Devices (Amendment etc.) (EU Exit) Regulations 2019, SI 2019/791, para 2.1.

4 Queen Elizabeth II, “Queen’s Speech December 19, 2019.”

5 The term ‘software’ was in fact only added to the definition of ‘medical device’ by an amendment in 2008, inserted by Art 1 of Directive 2007/47/EC OJ L 247/21. Recitals 6 and 20 of the 2007 Directive also illustrate a growing recognition of the issues associated with software. Elsewhere, some of us have written on the failure of legislation to grapple with software in this regard. See Downey, “Software as Medical Device”; Quigley, “Living in a Material World?” See further McHale, “Health Law, Brexit and Medical Devices.”

6 Melvin, “The Regulator’s View.” 351–6.

7 Medical Devices (Amendment etc.) (EU Exit) Regulations 2019/791; Human Medicines and Medical Devices (Amendment etc.) (EU Exit) Regulations 2019, SI 2019/1385.

8 GOV.UK, “MHRA Consultation EU Exit No-Deal.”

9 Medical Devices (Amendment etc.) (EU Exit) Regulations 2020, SI 2020/1478.

10 European Union (Withdrawal) Act 2018.

11 These were made using powers conferred by the *European Union (Withdrawal Act) 2018*, sch 4, s 8C, para 1(1)(ab) and sch 7, para 21, which confers powers to make regulations to implement the Northern Ireland Protocol.

12 See, for example, the foreword to the Department of Health and Social Care, Medical Technology Strategy, 3.

13 GOV.UK, “Innovative Devices Access Pathway.”

14 Medicines and Healthcare products Regulatory Agency, “New Regulatory Pathway.”

15 Accelerated Access Collaborative, “Innovative Devices Access Pathway (IDAP),” para 1.

16 Department for Business and Trade, “MedTech.”

17 HC Ninth Delegated Legislation Committee Deb November 26, 2020, cols 1–10.

18 For example, Lord Patel made clear his surprise at the MHRA guidance being published within days of the House of Lords second reading on the MMD Bill, which showed vast planned divergence between regulation for NI and GB, commenting that this had been published based on regulations that the Lords had not yet seen (HL Deb November 17, 2020, vol 807, cols 686–688GC; HL Deb November 19, 2020, vol 807, col 719GC). Baroness Penn’s response was that the statutory instruments on which this guidance was based had been laid before parliament on October 20 and were due to be debated later, but that they did not concern future issues relating to medical device regulation (HL Deb November 19, 2020, vol 807, col719GC). Thus, Peers were not alerted to the fact that these Regulations would remove alignment with the EU MDR and EU IVDR from the 2019 Regulations, and, somewhat misleadingly, were led to believe that the Regulations were not pertinent to the issues of future regulation.

19 Medicines and Medical Devices Act, s 15.

20 Interestingly, the framing here is similar to that in Preamble 1 in the EU MDR, which states the need for ‘a regulatory framework for medical devices which ensures high level of safety and health whilst supporting innovation’. Our thanks to Tom Melvin for pointing this out.

21 Medicines and Medical Devices Bill 90, s 12(2).

22 Medicines and Medical Devices HL Bill 154, s 14(2).

23 Medicines and Medical Devices HL Bill 154, s 14(2).

24 HL Deb October 26, 2020, vol 807, col 5GC (Baroness Thornton), col 10GC (Lord Heath).

25 See, for example, HL Deb October 26, 2020, vol 807, col 5GC (Baroness Thornton), col 6GC (Baroness Cumberlege) and col 11GC (Baroness Burt of Solihull). See also Dickson, “Lords Keep Up Pressure.”

26 Downey, “Increased Scrutiny.”

27 Medicines and Medical Devices Act 2021, s 16.

28 Medicines and Medical Devices Bill Deb June 23, 2020, col 4.

29 House of Lords Delegated Powers and Regulatory Reform Committee, Guidance for Departments, 5.

30 House of Lords Select Committee on the Constitution, Medicines and Medical Devices Bill, 2. See also the House of Lords Delegated Powers and Regulatory Reform Committee, Medicines and Medical Devices Bill.

31 See House of Commons, Tuesday June 23, 2020 Consideration of Bill (Report Stage), 3 (Alex Hancock); House of Lords, Bill 154-I, January 6 2021 Marshalled List of Amendments to be Moved on Report, 2 (Baroness Thornton, Baroness Joll and Lord Patel). Note that including a sunset clause was something recommended in written evidence submitted to parliament. See Quigley, Written Evidence, 3.

32 HL Deb October 19, 2020, vol 806, col 327GC (Lord Bethell).

33 Macleod, ‘The Independent Medicines and Medical Devices Safety Review.”

34 For a detailed account of this, see Macleod, “The Independent Medicines and Medical Devices Safety Review.”

35 Medicines and Medical Devices Act 2021, s 1. For further detail see Schedule 1 of the Act.

36 Medicines and Medical Devices Act 2021, s 19.

37 Signed April 10, 1998 by the UK and Irish Governments and political parties in Northern Ireland.

38 The Northern Ireland Protocol is a protocol to the EU–UK Withdrawal Agreement. The Windsor Framework was agreed between the UK and the EU on February 27, 2023 and implemented by agreement of the Withdrawal Agreement Joint Committee on March 24, 2023. See GOV.UK, “Windsor Framework – further detail publications.”

39 This is also true of regulations pertaining to human medicines and veterinary medicines (Northern Ireland Protocol art 5.4, para 21, Annex 2). For an in-depth analysis of the effect of Brexit and the Protocol on medicines, medical devices and equipment, see Hervey, Not What the Bus Promised, 110–129.

40 Medical Devices Regulation 2017/865, articles 61(4)–(5) & s 3, Annex XIV. See Medical Device Coordination Group, Clinical Evaluation.

41 See Hancher, “Revision of the Regulatory Framework,” 432; Heneghan, “Ongoing Problems”; Contardi, “Changes in the Medical Device’s Regulatory Framework,” 173.

42 See In Vitro Diagnostic Regulation 2017/866, Annex II. See also Reinikainen, IVD Classification Issues, 3.

43 Medicines and Healthcare products Regulatory Agency, “Timeframe for Accepting CE Marked Devices Extended.”

44 Medicines and Healthcare products Regulatory Agency, “Regulating Medical Devices.”

45 See Medical Devices Regulation 2002, SI 2002/618, regs 10–11, 24–25, and 36–37 (NI Interpretation).

46 See Medical Devices Regulation 2002, SI 2002/618, regs 13, 27, and 40 (NI Interpretation).

47 Medical Devices Regulation 2002, SI 2002/618, regs 10A, 24A, and 36A as inserted by Medical Devices (Amendment etc.) (EU Exit) Regulations 2020, SI 2020/1478, paras 3, 19, and 13, sch 1.

48 Medical Devices Regulation 2002, SI 2002/618, regs 10A(3), 24A(3), and 36A(3).

49 Medicines and Healthcare products Regulatory Agency, “Regulating Medical Devices.”

50 Medical Devices (Amendment) (Great Britain) Regulations 2023 No 627.

51 For the contrasting position in relation to the negative effect on the supply of medicines in NI, see Hervey, Not What the Bus Promised, 118–123.

52 Medical Devices Regulation 2002, SI 2002/618, reg 2A as inserted by Medical Devices (Amendment etc.) (EU Exit) Regulations 2019, SI 2019/791, reg 3 (4A) as inserted by Medical Devices (Amendment etc.) (EU Exit) Regulations 2020, SI 2020/1478, sch 2, para 6. The definition of a ‘qualifying Northern Ireland good’ has the same meaning as that in European Union (Withdrawal) Act 2018, s 8C(6) (as inserted by the *European Union (Withdrawal Agreement) Act 2020*, s2 1) which in turn simply provides a delegated power to a Minister to make regulations to define ‘Northern Ireland Good’. This has been established by the Definition of Qualifying Northern Ireland Goods (EU Exit) Regulations 2020.

53 Medicines and Healthcare products Regulatory Agency, “Regulating Medical Devices.” See also His Majesty's Revenue and Customs “Moving qualifying goods from Northern Ireland to the rest of the UK”.

54 Hervey, Not What the Bus Promised, 123. On this, see also Dayan, “Protocol Politics Mean Hard Times.”

55 Something that some of us noted in both briefing notes and written evidence to parliament during the passage of the Act. See Quigley, Briefing Note; Quigley, Written Evidence.

56 Legislation.gov.uk, “Title Search Results for Medical Devices.”

57 See House of Commons, June 10, 2020 Public Bill Committee, 8 (Philippa Whitford and Alex Hancock); House of Lords, Bill 154-I, January 6, 2021 Marshalled List of Amendments to be Moved on Report, 8 (Lord Patel, Baroness Thornton, Lord Kakkar and Lord MacKay of Clashfern). See also Quigley, Written Evidence, 3.

58 See, in particular, comments from Baroness Thornton and Lord Patel (Medicines and Medical Devices Bill Deb October 19, 2020, vol 806).

59 HL Deb October 19, 2020, vol 806, col 324GC.

60 Medicines and Medical Devices Act 2021, s 48.

61 See Quigley, Briefing Note, 2; Quigley, Written Evidence; HL Deb October 19,2020, vol 802, col 318GC (Baroness Thornton), col 31–3208GC (Lord Patel), col 323GC (Lord Kakkar), and col 325GC (Lord O’Shaughnessy); HL Deb January 12, 2021, vol 809, col 634 (Baroness Thornton), col 636–637GC (Lord Patel), col 637GC (Lord MacKay of Clashfern), col 638–639GC (Lord Kakkar), col 641GC (Lord Hunt of Kings Heath), and col 643GC (Baroness Jolly).

62 See HL Deb October 19, 2020, vol 802, col 324GC (Lord Lansley); HL Deb January 12, 2021, vol 809, col 638–639GC (Lord Kakkar) and col 639–641GC (Lord Lansley).

63 HL Deb January 12, 2021, vol 809, col 646GC (Lord Bethell).

64 In the recent consultation on the Law Commission’s 14th Programme of Law Reform, some of the present authors suggested that this should be undertaken. See Downey, “Responses to the MHRA Consultation.”

65 HC Ninth Delegated Legislation Committee, The Draft Medical Devices (Amendment etc.) (EU Exit) Regulations 2020, Deb November 26, 2020.

66 HC Ninth Delegated Legislation Committee, The Draft Medical Devices (Amendment etc.) (EU Exit) Regulations 2020, Deb November 26, 2020, col 4.

67 See Legislation.gov.uk, “Medical Device Regulations 2002.”

68 Heneghan, “Medical-Device Recalls.”

69 HC Deb March 2, 2020, vol 672, col 665 (Jonathan Ashworth MP).

70 Regulatory Horizons Council, Report on Medical Devices, 5.1.

71 For a detailed examination of the findings of the Review, see in this issue Macleod, “The Independent Medicines and Medical Devices Safety Review.”

72 Initially, Baroness Cumberlege tabled her own amendment to the Bill on this. However, this was later withdrawn in favour of a government amendment, which was accepted by all parties.

73 Quigley, “Integrating the Biological,” 301–302.

74 Department of Health and Social Care, “First Ever Patient Safety Commissioner.”

75 Moore, “A Missed Opportunity?”

76 Avery, “Retrospective Case Note Review.”

77 Mid Staffordshire NHS Foundation Trust, Public Inquiry Report.

78 Ockenden, Independent Maternity Review Final Report.

79 Moore, “A Missed Opportunity?”

80 Medicines and Medical Devices Act 2021, sch 1, para 4.

81 Moore, “Regulating Patient Safety,” 213.

82 HL Deb September 2, 2020, vol 805, col 372.

83 Fahy, “How will Brexit Affect Health?”; Fahy, “Impact on the NHS.” Leaving the EU also affects access to and participation in clinical research (much of which was funded by the EU) and access to the EU’s medical devices warning system and database (EUDAMED). For the sake of brevity, we do not discuss these here.

84 See Dayan, “Brexit Medicines Shortages.”

85 Regulatory Horizons Council, Report on Medical Devices.

86 Quigley, Briefing Note. See also Quigley, “Patient Safety Must Be the Priority.”

87 Medicines and Medical Devices Act 2021, s 15(2). The exact same wording was inserted for both medicines and veterinary medicines. Interestingly, this is similar to the wording of the objective in all EU legislation conferred by the Treaty on the Functioning of the European Union, art 168.

88 See section 2.2 in this article.

89 This refers to s 15(4) of the MMD Act; see also HL Deb January 12, 2021, vol 809, col 675GC and 711GC (Lord Bethell).

90 Medicine and Medical Devices Act, s 15(4).

91 Medicines and Healthcare products Regulatory Agency, Government Response to Consultation.

92 Medicines and Healthcare products Regulatory Agency, Consultation on Future Regulation, Introduction.

93 Medicines and Healthcare products Regulatory Agency, Government Response to Consultation, para 2.2.

94 Medicines and Healthcare products Regulatory Agency, Consultation on Future Regulation, para 2.3.

95 Our thanks to Tom Melvin for pointing this out.

96 Medicines and Healthcare products Regulatory Agency, Consultation on Future Regulation, ch 7.

97 Medical Devices Regulation 2017/865, art 63–66.

98 Melvin, “Orphan Medical Devices and Pediatric Cardiology.”

99 See Medical Device Coordination Group, Transition to MDR and IVDR. See also and BVMed and VDGH, Future Development, para 3.2.1–3.2.2.

100 Dayan, “Parallel, Divergent or Drifting?” 19.

101 Medicines and Healthcare products Regulatory Agency, Consultation on Future Regulation, para 31.11.

102 Medical Devices Regulation 2017/865, para. 3, Annex XIV.

103 See Medical Device Coordination Group, Clinical Evaluation; Holborow, “Claiming Equivalence.”

104 Medicines and Healthcare products Regulatory Agency, Consultation on Future Regulation, para 31.11.

105 Medicines and Healthcare products Regulatory Agency, Government Response to Consultation, para 31.2.

106 Food and Drug Administration, “CDRH Transparency.”

107 Li, “Regulating Artificial Intelligence.”

108 Medicines and Healthcare products Regulatory Agency, “Software and AI—Roadmap.”

109 Medicines and Healthcare products Regulatory Agency, Government Response to Consultation, para 19.2.

110 International Medical Device Regulators Forum UDI Working Group, “UDI Guidance.”

111 European Commission, “Unique Device Identifier.” See also Food and Drug Administration, “UDI Benefits.”

112 Nichols, “Ultimate Guide to UDI.”

113 GMDN Agency, “What is GMDN?”

114 Medicines and Healthcare products Regulatory Agency, Government Response to Consultation, para 18.2.

115 Medical Devices Regulation 2017/765, Art 26.

116 In Vitro Diagnostic Regulation 2017/766, Art 23.

117 See European Commission, European Medical Device Nomenclature (EMDN); Medical Device Coordination Group, FAQ on the European Medical Device Nomenclature (EMDN).

118 Directorate-General for Health and Food Safety, “The European Medical Device Nomenclature (EMDN)”.

119 Medicines and Healthcare products Regulatory Agency, Government Response to Consultation, para 19.2.

120 Our thanks to Tom Melvin for prompting us to think about this.

121 Food and Drug Administration, “Medical Device Single Audit Program.”

122 Medicines and Healthcare products Regulatory Agency, Government Response to Consultation, para 72.2.

123 Medical Device Coordination Group, Guidance on MDSAP Audit Reports Use.

124 Medicines and Healthcare products Regulatory Agency, Government Response to Consultation, para 72.2.

125 Medicines and Healthcare products Regulatory Agency, Government Response to Consultation, 8.

126 Medicines and Healthcare products Regulatory Agency, Government Response to Consultation, para 73.2.

127 Accelerated Access Collaborative, “Innovative Devices Access Pathway (IDAP),” para 3.

128 Medicines and Healthcare products Regulatory Agency, “Innovative Licensing and Access Pathway.”

129 Medicines and Healthcare products Regulatory Agency, “£10M from HM Treasury.”

130 Medicines and Healthcare products Regulatory Agency, Consultation on Future Regulation, Introduction.

131 Geographical health inequalities are already prevalent in the UK, with different levels of service available from different NHS Trusts. For more information about health inequalities, see Williams, “What are Health Inequalities?”

132 From the French: ‘plus ça change, plus c’est la même chose’. Downey, “Future of Medical Devices Legislation.”

133 Hervey, Not What the Bus Promised, 113.

134 Hervey, Not What the Bus Promised, 113.

135 Our thanks to one of the anonymous reviewers for prompting us to draw out our points here.

136 Hervey, Not What the Bus Promised, 125.

137 We say ‘new’, because although it is true that no longer being an EU Member State is new for the UK and that there are some genuinely new regulatory proposals pending, it is also true, as we have argued, that some of what is proposed is not particularly new and is very similar to what is contained in the EU MDR and EU IVDR and the UK’s 2019 EU Exit Regulations vis-à-vis medical devices.
